# Selected Indices to Identify Water-Stress-Tolerant Tropical Forage Grasses

**DOI:** 10.3390/plants11182444

**Published:** 2022-09-19

**Authors:** Alan Mario Zuffo, Fábio Steiner, Jorge González Aguilera, Rafael Felippe Ratke, Leandra Matos Barrozo, Ricardo Mezzomo, Adaniel Sousa dos Santos, Hebert Hernán Soto Gonzales, Pedro Arias Cubillas, Sheda Méndez Ancca

**Affiliations:** 1 Department of Agronomy, State University of Maranhão, Balsas, MA 65800-000, Brazil; 2 Department of Crop Science, State University of Mato Grosso do Sul, Cassilândia, MS 79540-000, Brazil; 3 Department of Agronomy, Federal University of Mato Grosso do Sul, Chapadão do Sul, MS 79560-000, Brazil; 4 Department of Plant Sciences, Federal University of Piauí, Bom Jesus, PI 64900-000, Brazil; 5 Escuela Profesional de Ingeniería Ambiental, Universidad Nacional de Moquegua (UNAM), Ilo 18601, Peru; 6 Escuela de Posgrado-Doctorado en Ciencias Ambientales, Universidad Nacional Jorge Basadre Grohmann (UNJBG), Tacna 23001, Peru; 7 Escuela Profesional de Ingeniería Pesquera, Universidad Nacional de Moquegua (UNAM), Ilo 18601, Peru

**Keywords:** soil water regime, stress tolerance indices, forage yield, *Panicum maximum*, *Urochloa* sp.

## Abstract

Periods of soil water stress have been recurrent in the Cerrado region and have become a growing concern for Brazilian tropical pasture areas. Thus, the search for forage grasses more tolerant to water stress has intensified recently in order to promote more sustainable livestock. In a greenhouse experiment, the degree of water stress tolerance of nine tropical forage grass cultivars was studied under different soil water regimes. The investigation followed a 9 × 3 factorial design in four randomized blocks. Nine cultivars from five species of perennial forage grasses were tested: *Urochloa brizantha* (‘BRS Piatã’, ‘Marandu’, and ‘Xaraés’), *Panicum maximum* (‘Aruana’, ‘Mombaça’, and ‘Tanzânia’), *Pennisetum glaucum* (‘ADR 300’), *Urochloa ruziziensis* (‘Comum’), and *Paspalum atratum* (‘Pojuca’). These cultivars were grown in pots under three soil water regimes (high soil water regime—HSW (non-stressful condition), middle soil water regime—MSW (moderate water stress), and low soil water regime—LSW (severe water stress)). Plants were exposed to soil water stress for 25 days during the tillering and stalk elongation phases. Twelve tolerance indices, including tolerance index (TOL), mean production (MP), yield stability index (YSI), drought resistance index (DI), stress tolerance index (STI), geometric mean production (GMP), yield index (YI), modified stress tolerance (k_1_STI and k_2_STI), stress susceptibility percentage index (SSPI), abiotic tolerance index (ATI), and harmonic mean (HM), were calculated based on shoot biomass production under non-stressful (Y_P_) and stressful (Y_S_) conditions. Soil water stress decreased leaf area, plant height, tillering capacity, root volume, and shoot and root dry matter production in most cultivars, with varying degrees of reduction among tropical forage grasses. Based on shoot biomass production under controlled greenhouse conditions, the most water-stress-tolerant cultivars were *P. maximum* cv. Mombaça and cv. Tanzânia under the MSW regime and *P. maximum* cv. Aruana and cv. Mombaça under the LSW regime. *P. maximum* cv. Mombaça has greater adaptability and stability of shoot biomass production when grown under greenhouse conditions and subjected to soil water stress. Therefore, this forage grass should be tested under field conditions to confirm its forage production potential for cultivation in tropical regions with the occurrence of water stress. The MP, DI, STI, GMP, YI, k_2_STI, and HM tolerance indices were the most suitable for identifying forage grass cultivars with greater water stress tolerance and a high potential for shoot biomass production under LSW regime.

## 1. Introduction

The large territorial extension and the edaphoclimatic conditions in Brazil are fundamental elements for the country to have an expressive development of its livestock and agriculture activities. Brazil is one of the world’s largest producers and exporters of animal food, as well as the origin of a great number of plant species. The country has the world’s second-largest cattle herd, with 252 million head, and its production is based on grass pastures [1]. Brazil has a pasture area of approximately 172 million hectares, of which 102 million hectares are cultivated with forage plants and 70 million hectares are native pastures [2].

Because national meat and milk production are highly dependent on the natural feeding of grass and/or legume pastures, the quality of pastures is essential for Brazilian livestock activity [3]. In addition, many Brazilian producers are diversifying their agricultural production systems by cultivating tropical forage grasses in the off-season of cash crops to produce forage for cattle in the autumn/winter and straw in the spring for the agricultural production system [4]. This production system, called the Integrated Crop-Livestock System (ICLS), is an important strategy for producing quality pasture at a time of low rainfall in the Brazilian Cerrado region. However, this dry season during southern winter poses many challenges to Brazilian livestock activity, especially concerning the supply of quality pasture. Therefore, studies that evaluate and identify genotypes of forage grasses with greater water stress tolerance are essential for boosting animal production systems.

The most important tropical forage grasses used in livestock and agriculture systems in the Cerrado region are species of the genera *Urochloa, Cynodon, Panicum, Paspalum*, and *Pennisetum*. These forage grasses are currently the basis of food for meat and milk cattle, mainly due to their excellent nutritional quality and adequate adaptation to Brazilian production systems [5]. However, each forage grass species or cultivar has a distinct biomass production potential that depends on morphological and genetic characteristics. Pearl millet (*Pennisetum glaucum* (L.) R. Br.), palisade grass (*Urochloa brizantha* (Hochst. Ex A. Rich.) R.D. Webster), and ruzigrass (*U. ruziziensis* (R. Germ. & C.M. Evrard) Crins) have been cultivated due to their high biomass production capacity [6]. Improvement of forage grasses’ productivity in quantity, as well as its quality, would have a significant impact on livestock production. Furthermore, palisade grass and ruzigrass have also been described as water-stress-tolerant forage grasses [7]. Therefore, looking for species and/or cultivars tolerant to water stress is of fundamental importance to mitigate the negative impacts of low soil water availability and increase forage production in the dry off-season [3,5,8], a period of the year with a deficient supply of pasture for cattle in Brazil.

Soil water stress results in a dramatic decline in leaf expansion rate and photosynthesis rate, which inhibits plant development and reduces the biomass production of forage grasses [8,9], especially by causing changes in root growth, leaf initiation rate, nutrient uptake, and carbohydrate metabolism [10,11,12,13]. However, the response of forage grasses to water stress depends on genotype, plant developmental stage, severity, and duration of the water stress period [6,7,8]. Generally, forage grasses are more susceptible to soil water stress during the tillering and stalk elongation phases, and stalk and leaf growth are more affected than other plant organs [9,12]. Typical effects of water stress on forage grasses include leaf rolling, stomatal closure, stalk and leaf growth inhibition, early leaf senescence, reduced leaf area, and reduced biomass production [8,11]. Some of these responses are part of the plant’s strategies that aim to mitigate the adverse effects of low soil water availability and, therefore, constitute water stress tolerance mechanisms.

Water stress tolerance refers to the degree to which a plant is adapted to low soil water availability or drought conditions [14]. Forage plants are subject to periods of water stress during their growth and development phase and must adapt to these adverse conditions. Thus, plants in adverse environments have the ability to endure water stress through certain biochemical or morphological adaptations and avoidance of cell injury [9,12,15,16].

Understanding forage grass responses to water stress periods is essential to pasture biomass productivity mainly because the periodic and repeated water shortage have increasingly concerned Brazilian tropical grasslands [5]. Thus, identifying and understanding water stress tolerance mechanisms are fundamental factors for developing tolerant forage grass cultivars. The relative performance of forage grass biomass production under non-stressful and stressful conditions (i.e., high and low soil water availability) seems to be the starting point for identifying species with greater water stress tolerance [17]. Therefore, the main conditions that must be considered when determining water-stress-tolerant and water-stress-sensitive cultivars are cropping under non-stressful and stressful conditions with high and low soil water availability, respectively [18,19]. However, identifying water-stress-tolerant genotypes is not an easy task because the productive performance of forage grasses is the result of a genotypic expression modulated by continuous interaction with the growing environment [8].

Some studies have proposed using different selection indices to evaluate and identify water-stress-tolerant genotypes. Some of these selection indices were used to assess genetic differences in genotypes of sorghum [18], soybeans [19], maize [20], wheat [21,22], sunflower [23], and common beans [24]. However, these studies for tropical forage grasses are still unknown. Therefore, our research constitutes the first report on selection indices to assess the degree of water stress tolerance of the main forage grasses used in the Brazilian Cerrado region. This information will help Brazilian farmers choose the best forage cultivars to be planted in areas subject to soil water stress.

This study aimed to determine the degree of water stress tolerance of nine cultivars of tropical forage grasses grown under different soil water regimes.

## 2. Results and Discussion

### 2.1. Analysis of Variance

Analysis of variance revealed that the effect of soil water regime was significant (*p* < 0.01) on all forage grass growth traits. The interaction between soil water regime and cultivars showed a significant effect (*p* < 0.05) on all plant growth traits except for the number of tillers and shoot dry matter (Table 1). The significant interaction between the main effects of cultivars and soil water regimes on most morphological traits indicates that the forage grasses have distinct responses when exposed to soil water availability levels.

### 2.2. Morphological Responses of Forage Grasses to Water Stress

Soil water stress resulted in a lower growth rate in plant height of *U. brizantha* cv. BRS Piatã, *P. glaucum* cv. ADR 300 and *P. maximum* cv. Mombaça compared to high soil water regime (Table 2). The plant height of *P. maximum* cv. Mombaça, *U. brizantha* cv. BRS Piatã and *P. glaucum* cv. ADR 300 was 22%, 28%, and 44% lower in plants grown under a low soil water (LSW) regime when compared to plants under a high soil water (HSW) regime. However, soil water regimes did not inhibit the height growth rate of other forage grasses.

Petter et al. [6] reported that the growth rate of *U. brizantha* cv. Xaraés, *U. ruziziensis* cv. Comum and *P. glaucum* cv. ADR 7010 was not negatively affected by plant exposure to water stress. However, Zuffo et al. [25] showed that soil water stress inhibited the height growth rate of *U. brizantha* cv. BRS Piatã and *P. glaucum* cv. ADR 300. These results show that the adverse effects of water stress on the height growth rate of forage grasses are still inconsistent and depend on the grass development stage at which water stress occurs. Soil water stress imposed during the initial growth stage has a more significant negative impact on plant height than when imposed during the grass tillering stage [16].

The highest plant height under different soil water regimes was observed for *P. glaucum* cv. ADR 300; however, the plant height was similar to *P. maximum* cv. Aruana under an LSW regime (Table 2). These results are associated with the growth habits of forage grass cultivars. The *P. glaucum* is more extensive and has an erect growth habit, while *U. brizantha* and *P. maximum* plants are smaller, have a more tufted growth habit, and have a greater number of tillers [6,26].

Water stress significantly reduced (*p* < 0.05) the number of leaves of *U. ruziziensis* cv. Comum, *P. maximum* cv. Aruana, *P. maximum* cv. Mombaça, and *P. Atratum* cv. Pojuca, while the other forage grasses did not significantly reduce the number of leaves when grown under different soil water regimes (Table 2). Under an HSW regime, the highest number of leaves was observed for *P. atratum* cv. Pojuca, while under MSW and LSW regimes, the highest number of leaves was obtained in *U. ruziziensis* cv. Comum and *P. atratum* cv. Pojuca plants. Petter et al. [6] also reported a higher number of leaves in *U. ruziziensis* plants exposed to soil water stress. These results indicate that even the plants of *U. ruziziensis* have a significant reduction in the number of leaves when subjected to soil water stress; this species can maintain a high number of leaves under LSW regimes. The lower emergence of new leaves under water stress conditions has been considered a plant strategy to reduce the transpiration rate and increase water use efficiency [15]. However, this water stress tolerance strategy is conditioned on the specific response of the genotype [9]. The lower number of leaves has been a response of forage grasses to ensure their survival to relatively long periods of low soil water availability [8].

Leaf area was significantly (*p* < 0.05) smaller under an LSW regime for all forage grass cultivars except for *P. glaucum* cv. ADR 300 and *P. atratum* cv. Pojuca (Table 2). Under an LSW regime, the leaf area reduction of forage grasses ranged from 45% to 62% compared to plants under an HSW regime. Leaf area reduction in three forage grass cultivars (*U. brizantha* cv. BRS Piatã, *U. brizantha* cv. Marandu, *P. glaucum* cv. ADR 300) exposed to water stress conditions were also reported by Zuffo et al. [25]. The reduction in leaf area has been reported as a typical response of forage grasses when exposed to soil water stress [8,9,10,11,12]. One of the first processes affected in response to decreased soil water availability is cell expansion, a highly dependent process of turgidity in plants. However, with the advancement of soil water stress, other physiological processes are negatively affected, with direct effects on the photoassimilates accumulated by the forage grasses, reduction in the carbon assimilation rate, and relative growth rate [8,9]. As a result of these effects, there is a reduction in leaf area and biomass production. The reduction in leaf area occurs as a defense reaction of plants to water stress, reducing the transpiration rate and, consequently, water loss to the atmosphere [15].

Soil water regimes did not alter the root dry matter accumulation of *U. brizantha* cv. Xaraés, *P. glaucum* cv. ADR 300 and *P. atratum* cv. Pojuca, while the root dry matter accumulation of the other forage grasses was significantly lower when grown under water stress, especially under an LSW regime (Table 3). Under an HSW regime, root dry matter was higher for *U. brizantha* cv. BRS Piatã, *U. brizantha* cv. Marandu, *U. ruziziensis* cv. Comum, *P. maximum* cv. Aruana, *P. maximum* cv. Mombasa and *P. maximum* cv. Tanzania. However, when forage grass cultivars were grown under an LSW regime, there were no significant differences in root dry matter production (Table 3). The lower production of root dry matter of forage grasses under water stress conditions was also reported by Petter et al. [6] and Fariaszewska et al. [9]. Under soil water stress, plants reveal mechanisms to combat cellular tissue dehydration. The decrease in soil water availability causes an increase in the synthesis of abscisic acid (ABA) and stress proteins, which protect cell membranes and participate in osmoregulation [8]. The increase in the concentration of ABA in the cells reduces the transpiration rate by closing the stomata and results in greater water use efficiency [9]. In addition, abscisic acid inhibits shoot growth but simultaneously stimulates root growth and development, which essentially helps to overcome stress [27]. However, this stimulus in root growth caused by the higher concentration of ABA under water stress conditions has been commonly reported under field conditions [8,12]. In pot experiments, as in this study, the growth of the plant root system was limited by the soil volume in the pot.

Total dry matter accumulation and root volume were significantly lower (*p* < 0.05) under water stress conditions for all forage grass cultivars (Table 3). Under HSW regime, *P. maximum* cv. Mombaça plants have greater total dry matter accumulation and greater root volume than other forage grasses. Under soil water stress, plants of *U. ruziziensis* cv. Comum, *P. maximum* cv. Aruana, *P. maximum* cv. Mombaça, and *P. maximum* cv. Tanzânia had a greater total dry matter production, except for *P. maximum* cv. Aruana under an MSW regime (Table 3). Under water stress conditions, there was no significant difference in root volume value between forage grass cultivars, except for *P. glaucum* cv. ADR 300 and *P. atratum* cv. Pojuca under MSW, which had lower root volume than other forage grasses. The lower total dry matter production and root volume of plants exposed to soil water stress is a consequence of plant adaptation mechanisms to avoid excessive water loss [11,12], as well as the adverse effects of water stress on plant physiological metabolism, especially on the photosynthetic activity of the plants [9]. Under water stress conditions, the rate of photosynthesis decreases, which is related to a decrease in rubisco activity, a reduction in stomatal conductance, and reduced availability of CO_2_ [8].

Plants of *U. ruziziensis* vc. Comum has a higher tiller emission rate, while plants of *U. ruziziensis* cv. Comum, *P. maximum* cv. Aruana, *P. maximum* cv. Mombaça, and *P. maximum* cv. Tanzânia had a higher production of shoot dry matter (Table 4). Tillers are very important to understanding forage grass growth and regrowth. Tillers are new grass shoots made up of successive segments called phytomers. The tillering rate of grass species is controlled by the emergence rate of phytomers, genetic characteristics, and plant density in the field [5]. Thus, shoot dry matter production results from accumulated phytomers per stem and the stem density per area [11,16].

The LSW regime inhibited the tiller emission of forage grasses (Table 4). Shoot dry matter production was drastically reduced when grasses were exposed to water stress conditions. The lower tillering rate and shoot dry matter accumulation of *U. brizantha* cv. BRS Piatã and P. glaucum cv. ADR 300 under water stress conditions was also reported by Zuffo et al. [25]. When water stress occurs at the initial stage of grass development, the reduction in stomatal conductance and photosynthesis rate results in lower plant tillering potential and lower shoot dry matter production [9], which results in significant forage production losses [8]. According to Fonseca and Martuscello [5], the main morphological characteristics that directly affect the forage production potential are the number of tillers, the number of leaves, and leaf size.

### 2.3. Interrelationship between Morphological Traits and Forage Grass Cultivars

**Figure 1:**
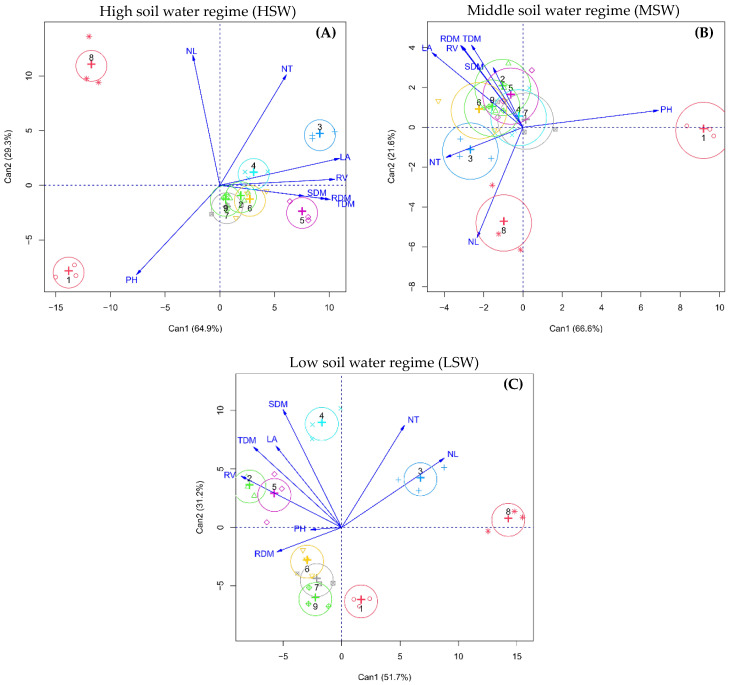
Canonical correlation analysis (CCA) between the morphological traits and forage grass cultivars when grown under well-irrigated control conditions (**A**) or exposed to moderate water stress (**B**) and severe water stress (**C**). The blue lines show the canonical correlation between the centroids of the first pair of canonical variates and the linear tendency line. Abbreviations: PH: plant height; NT: number of tillers; NL: number of leaves; LA: leaf area; SDM: shoot dry matter; RDM: root dry matter; TDM: total dry matter; RV: root volume. (T1) *P. glaucum* cv. ADR 300; (T2) *P. maximum* cv. Tanzânia; (T3) *U. ruziziensis* cv. Comum; (T4) *P. maximum* cv. Aruana; (T5) *P. maximum* cv. Mombaça; (T6) *U. brizantha* cv. Marandu; (T7) *U. brizantha* cv. BRS Piatã; (T8) *P. atratum* cv. Pojuca; and (T9) *U. brizantha* cv. Xaraés.

Canonical correlation analysis was used to verify the contribution of each dependent variable measured in the tropical forage grasses as affected by soil water regimes (Figure 1). For scores to be represented in a two-dimensional graph, the percentage of retained variance must be higher than 80% [28]. In this study, variances accumulated in the two main canonical variables were 94.2%, 88.2%, and 82.9%, respectively, for each graph (Figure 1A–C), allowing an accurate interpretation.

Under the HSW regime, an angle (between vectors) less than 90° indicates a positive correlation between the dependent variable plant height (PH) with the *P. glaucum* cv. ADR 300 (T1); number of leaves (NL) with *P. atratum* cv. Pojuca (T8); and the number of tillers, leaf area, shoot dry matter, root dry matter, total dry matter, and root volume with *P. maximum* cv. Tanzania (T2), *U. ruziziensis* cv. Comum (T3), *P. maximum* cv. Aruana (T4), *P. maximum* cv. Mombaça (T5), *U. brizantha* cv. Marandu (T6), *U. brizantha* cv. BRS Piatã (T7), and *U. brizantha* cv. Xaraés (T9) (Figure 1A).

Under the MSW regime, there was a positive correlation between plant height and *P. glaucum* cv. ADR 300 (T1); the number of leaves and tillers with *U. ruziziensis* cv. Comum plants (T3) and *P. atratum* cv. Pojuca (T8); and leaf area, root volume and shoot, root and total dry matter with *P. maximum* cv. Tanzânia (T2), *P. maximum* cv. Aruana (T4), *P. maximum* cv. Mombaça (T5), *U. brizantha* cv. BRS Piatã (T7) and *U. brizantha* cv. Xaraés (T9) (Figure 1B).

Under the LSW regime, there was a positive correlation between the number of leaves and tillers with *U. ruziziensis* cv. Comum (T3) and *P. atratum* cv. Pojuca (T8); plant height and root dry matter with *U. brizantha* cv. Marandu (T6), *U. brizantha* cv. BRS Piatã (T7) and *U. brizantha* cv. Xaraés (T9); and leaf area, root volume, shoot, and total dry matter with *P. maximum* cv. Tanzânia (T2), *P. maximum* cv. Aruana (T4) and *P. maximum* cv. Mombaça (T5) (Figure 1C).

The greater or lesser negative impact of soil water stress on the growth and development of forage grasses is determined by the genetic traits of the genotype’s tolerance when exposed to water stress conditions. Each forage grass has distinct morphological characteristics that can be modified by the pasture’s production environment and technical management. However, this modification of the morphological traits of grasses is limited by the phenotypic plasticity of the genotype [12]. Therefore, the cultivation environment of forage grasses results in distinct gradual, reversible changes in the morphogenic and structural characteristics of the plants [29].

The results shown in Figure 1 indicate that under non-stressful or stressful conditions, forage grasses of the species *P. maximum* have a greater capacity to produce leaf area, root volume, shoot, and total dry matter. Therefore, when the farmer aims at greater forage production, cultivars Aruana, Mombaça, and Tanzânia of *P. maximum* are excellent option for cattle feeding. Under the LSW regime, grasses of the species *U. brizantha* have a greater capacity to produce root dry matter; however, the highest root volume under LSW was observed for *P. maximum* cultivars. *U. ruziziensis* plants have a higher tillering potential under non-stressful and stressful conditions. Therefore, it can be seen that each forage grass cultivar has its intrinsic characteristics and directs the accumulation of photoassimilates to different drains, either for the growth of the stem, leaves, roots, or tillers.

The pattern of dry matter allocation among different plant organs can change throughout the plant development stages, especially when exposed to stressful environmental conditions. However, this pattern of photoassimilate allocation is essential to optimize crop growth and development under stressful conditions. This is because the pattern of photoassimilate allocation can affect plants’ competitive and adaptive capacity and their responses to the stresses imposed by the cultivation environment [29]. The pattern of dry matter allocation in forage grasses is directly related to the optimization of capturing the scarcest resources from the cultivation environment. Under non-stressful conditions, grasses can allocate more photoassimilates to leaves to increase plants’ light energy uptake and photosynthetic rate and increase forage production. On the other hand, grasses can allocate more photoassimilates to the roots under water stress conditions to improve water and nutrient uptake when soil water availability is low or limited [12].

### 2.4. Water Stress Tolerance Indices

The highest shoot biomass production under HSW regime (Y_P_) was obtained for *P. maximum* cv. Aruana, *P. maximum* cv. Mombaça, and *P. maximum* cv. Tanzânia (Table 5). Under the MSW regime, the nine forage grass cultivars were grouped into the same group based on shoot biomass production (Y_S_) and TOL, YSI, DI, YI, k_2_STI, SSPI, and ATI indices. These results indicate that these tolerance indices did not effectively differentiate the water stress tolerance levels of forage grass cultivars exposed to moderate water stress conditions. Menezes et al. [18] also reported that the TOL and YSI indices did not differentiate water-stress-tolerant grain sorghum genotypes adequately. On the other hand, the MP, STI, GMP, and HM indices classified the forage grass cultivars into two tolerance groups, and the plants of *U. ruziziensis* cv. Comum, *P. maximum* cv. Aruana, *P. maximum* cv. Mombaça, and *P. maximum* cv. Tanzânia belonged to the group with the highest values of water stress tolerance indices (Table 5).

Under LSW regime, the TOL, YSI, and SSPI indices were not efficient in differentiating the water stress tolerance level of the nine forage grass cultivars (Table 5). On the other hand, the MP, GMP, and k_2_STI indices separated the forage grass cultivars into three tolerance groups. In contrast, the DI, STI, YI, and HM indices divided the forage grass cultivars into four tolerance groups (Table 5). These results indicate that these tolerance indices were the most sensitive to differentiate forage grass cultivars regarding water stress tolerance levels. Naghavi et al. [20] showed that the STI, YI, SSPI, k_1_STI, and k_2_STI indices were the most suitable to identify water-stress-tolerant maize genotypes. Cabral et al. [19] reported that the MP, STI, GMP, and HM tolerance indices are the most appropriate to identify water-stress-tolerant soybean cultivars. Sánchez-Reinoso et al. [24] reported that only the SSPI index effectively identified water-stress-tolerant bean genotypes.

### 2.5. Interrelationship between Biomass Production and Water Stress Tolerance Indices

**Figure 2:**
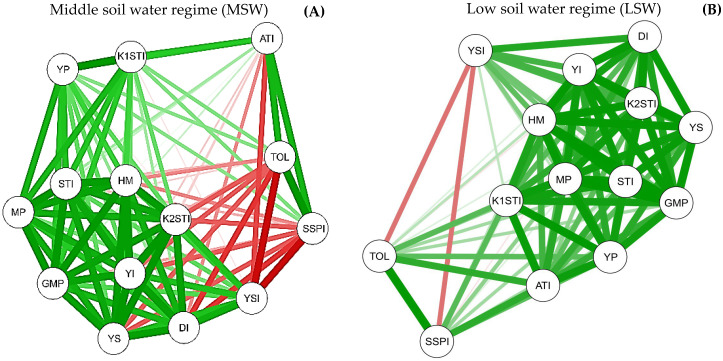
Correlation networks illustrate the most significant Pearson correlations between the shoot biomass production (Y_P_ and Y_S_) and water stress tolerance indices of tropical forage grasses grown under MSW (**A**) and LSW (**B**) regime. Thicker and green lines represent the highest positive correlations (threshold set at 0.6 and *p* values < 0.05). Thicker and red lines represent the highest negative correlations (threshold set at 0.6 and *p* values < 0.05). TOL: Tolerance, MP: Mean productivity, YSI: Yield stability index, DI: Drought resistance index, STI: GMP: Stress tolerance index, GMP: Geometric mean productivity, YI: Yield index, k_1_STI: Modified stress tolerance (k1), k_2_STI: Modified stress tolerance (k2), SSPI: Stress susceptibility percentage index, ATI: Abiotic tolerance index, HM: Harmonic mean.

A network diagram was constructed based on the shoot biomass production of forage grasses exposed to non-stressful and stressful conditions and on all stress tolerance indices and their respective correlations (Figure 2). The correlation network diagram shows the interactions between all the water stress tolerance indices with the shoot biomass production of the grasses. Positive and highly significant correlations were detected between water stress tolerance indices and shoot biomass production of forage plants grown under MSW (Figure 2A) or LSW (Figure 2B) regimes.

Under MSW regime, positive and significant correlations were detected between shoot biomass production under nonstressful conditions (Y_P_) with all stress tolerance indices; ATI with TOL, SSPI, k_1_STI, MP, GMP; k_1_STI with all tolerance tolerances except YSI. Negative and significant correlations were detected between shoot biomass production under stressful conditions (Y_S_) with SSPI, TOL, and ATI; between YSI and TOL, SSPI, and ATI; between TOL and MP, YI, STI, HM; between k_2_STI and SSPI; and between ATI and k_2_STI and YI (Figure 2A).

Under LSW regime, positive and significant correlations were detected between shoot biomass production under nonstressful (Y_P_) and stressful conditions (Y_S_) with all water stress tolerance indices. Negative and significant correlations were detected between the YSI with the SSPI and TOL indices (Figure 2B).

Discrimination of the water index tolerance level of the nine forage grass cultivars based on only one criterion or tolerance index can be contradictory (Table 5). Therefore, forage grasses should be differentiated and separated into different water stress tolerance levels based on all tolerance indices [20]. The ranking method has been used to classify crop genotypes into different water stress tolerance levels [21]. The ranking score of the nine forage grass cultivars in each of the 12 water stress tolerance indices under MSW and LSW regimes is shown in Table 6.

### 2.6. Ranking

Considering all water stress tolerance indices, the cultivars *P. maximum* cv. Mombaça and *P. maximum* cv. Tanzânia had the highest stress tolerance indices (Table 5) and the best-ranking scores under the MSW regime (Table 6). Therefore, these two forage grass cultivars were identified as tolerant to moderate water stress. The cultivars *U. brizantha* cv. Marandu, U. ruziziensis cv. Comum, and *P. maximum* cv. Aruana were identified as moderately tolerant to moderate water stress, while *P. glaucum* cv. ADR 300 and *P. atratum* cv. Pojuca was the most sensitive cultivar to moderate water stress (Table 6).

Under LSW regime, the cultivars *P. maximum* cv. Aruana and *P. maximum* cv. Mombaça were classified as water-stress-tolerant, whereas the cultivars *U. ruziziensis* cv. Comum and *P. maximum* cv. Tanzânia were classified as moderately tolerant to severe water stress (Table 6). These results suggest that the cultivation of *P. maximum* cultivars ‘Aruana’, ‘Mombaça’, and ‘Tanzânia’ is an excellent option for feeding cattle during the dry season in Brazil since these forage grass cultivars were identified as tolerant to water stress and have a high capacity for forage production in low soil water availability conditions. Indeed, using tolerant cultivars in areas subject to water stress is the best solution to face the predicted climate changes in the coming years and decades.

Fonseca and Martuscello [5] reported that forage grasses belonging to the species *P. maximum* have a high potential for forage yield response to stressful environmental conditions, confirming the results of this study. Some studies with wheat [30], canola [31], and sugarcane [10] suggest that the degree of water stress tolerance is related to the ability of plants to uptake and accumulate mineral nutrients when exposed to low soil water availability conditions. In general, water stress tolerance is the characteristic of a plant species or cultivar to adapt to growing environments with low water availability. Under these stressful conditions, the extension of the root system has been a fundamental morphological characteristic to improve the ability of plants to extract water and nutrients from the soil [12,16].

Plants of *P. atratum* cv. Pojuca were identified as sensitive to water stress under MSW and LSW regimes (Table 6). Therefore, the cultivation of this forage cultivar during the dry season in tropical regions of Brazil should not be recommended due to its low capacity to produce shoot biomass in soil water stress conditions.

## 3. Materials and Methods

### 3.1. Plant Growth Conditions

The experiment was conducted at Cassilândia, Mato Grosso do Sul, Brazil (19°05′29′′ S and 51°48′50′′ W, and altitude of 540 m) from May to August 2019, in 12 L plastic pots in a smart greenhouse with an automatic climate control system. The temperature and relative humidity inside the greenhouse were maintained at 26 °C (±2 °C) and 70% (±4%), respectively, during the trial period. The photosynthetic photon flux density (PPFD) inside the greenhouse, measured daily at midday (±12:00 h) with an Apogee MQ-500 quantum sensor, was 982 μmol m^−2^ s^−1^ (±238 μmol m^−2^ s^−1^).

The soil used in the experiment was a typic Quartzipsamment (or Neossolo Quartzarênico Órtico latossólico) collected from the 0.0–0.30 m layer in a Cerrado native pasture area with 180 g kg^−1^ of clay, 70 g kg^−1^ of silt, and 750 g kg^−1^ of sand. The occurrence of Quartzipsamments in the eastern region of Mato Grosso do Sul state is common. This soil class has no restrictions for the use and management of agricultural soil [32].

The chemical analysis showed that the soil used in this research has a low acidity level and high fertility level, which allowed the adequate availability of nutrients for forage plants. The soil chemical analysis reported the following results: pH in CaCl_2_ = 5.6; 20 g kg^−1^ of organic matter; 12 mg dm^−3^ of P (Mehlich^-1^); 3.70 cmol_c_ dm^−3^ of Ca^2+^; 1.60 cmol_c_ dm^−3^ of Mg^2+^; 0.22 cmol_c_ dm^−3^ of K^+^; 5.80 cmol_c_ dm^−3^ of CEC; 68% of base saturation; 1.8 mg dm^−3^ of Cu^2+^; 1.5 mg dm^−3^ of Zn^2+^; 73 mg dm^−3^ of Fe^2+^; and 13 mg dm^−3^ of Mn^2+^. All the soil chemical properties were analyzed according to Brazilian Agricultural Research Corporation standard methods described by Teixeira et al. [33].

The field capacity was measured under free-draining conditions using a water content decrease rate of 0.1 g kg^−1^ day^−1^, as proposed by Casaroli and Lier [34], and the soil volumetric water moisture content (VWC) at pot field capacity (FC) was 218 g kg^−1^.

The soil was placed in 12 L plastic pots and fertilized with 50 mg kg^−1^ of N (urea), 300 mg kg^−1^ of P (simple superphosphate), 150 mg kg^−1^ of K (potassium chloride), 30 mg dm^−3^ of S (gypsum), 2 mg kg^−1^ of Cu (copper sulfate), and 2 mg kg^−1^ of Zn (zinc sulfate). Fertilizers were incorporated into the entire soil volume of the pots three days before sowing the forage plants. Each plastic pot was filled with 14 kg (±10 dm^3^) of air-dried soil and sieved with a 5.0 mm mesh.

### 3.2. Experimental Design and Treatments

The experiment was arranged in a completely randomized block design in a 3 × 9 factorial arrangement with four replicates. Treatments consisted of three soil water regimes (high soil water regime—HSW (non-stressful condition), middle soil water regime—MSW (moderate water stress), and low soil water regime—LSW (severe water stress)) and nine cultivars of tropical forage grasses (*Urochloa brizantha* cv. BRS Piatã, *U. brizantha* cv. Marandu, *U. brizantha* cv. Xaraés, *U. ruziziensis* cv. Comum, *Pennisetum glaucum* cv. ADR 300, *Panicum maximum* cv. Aruana, *P. maximum* cv. Mombaça, *P. maximum* cv. Tanzânia, *Paspalum atratum* cv. Pojuca). Some of the morphological and agronomic characteristics of the tropical forage grasses used in this study are shown in Table 7.

Water stress treatments were applied for 25 days during the grass tillering and stalk elongation phases. Moderate and severe water stresses were achieved by simply changing the percentage volume of soil field capacity moisture in the pots, as proposed by Imakumbili [35]. This methodology often achieves severe water stress by maintaining the soil VWC in pots between 20% and 30% FC. An FC of 20% is acceptable for fine-textured soils, such as clayey soils, whereas an FC of 30% is suitable for coarse-textured soils, such as sandy soils. Moderate water stress is often achieved by maintaining soil VWC in pots at 60% FC in both coarse- and fine-textured soils, and 100% of FC will keep plants in pots under non-stressful conditions. The soil used in this study was a medium texture soil (180 g kg^−^^1^ of clay); therefore, the soil VWC in the pots was maintained at 60% and 25% of FC, respectively, for plants exposed to moderate water stress (middle soil water regime) or severe water stress (low soil water regime).

### 3.3. Plant Material and Irrigation

Seeds of nine tropical forage cultivars, three commercial cultivars of *Urochloa brizantha* (Hochst. Ex A. Rich.) R.D. Webster (‘BRS Piatã’, ‘Marandu’, and ‘Xaraés’), three commercial cultivars of *Panicum maximum* Jacq. (‘Aruana’, ‘Mombaça’, and ‘Tanzânia’), one commercial cultivar of *Pennisetum glaucum* (L.) R. Br. (‘ADR 300’), one commercial cultivar of *Urochloa ruziziensis* (R. Germ. & C.M. Evrard) Crins (‘Comum’), and a commercial cultivar of *Paspalum atratum* Swallen (‘Pojuca’) were sown on 8 May 2019 in 12 L pots. Ten seeds were sown at 2.0 cm depth, and five days after emergence, seedlings were thinned to two plants per pot. All plants were fertilized 30 days after emergence with 80 mg kg^−^^1^ of N via urea solution.

Until 40 days after sowing, the soil VWC was maintained at FC (218 g kg^−^^1^) with daily irrigation. Subsequently, the experiment was divided into three groups of soil water regimes [HSW (100% of FC), MSW (60% of FC), and LSW (25% of FC)]. When initiating water stress treatments, severely and moderately stressed pots were not irrigated for 9 and 6 days until the soil VWC in the pots had dropped to a point slightly below 25% and 60% of FC, respectively. Posteriorly, the soil VWC was maintained at these water stress levels for 25 days. Soil water availability in pots was controlled daily at 9:00 a.m. and 3:00 pm using the gravimetric method [35], and the soil VWC was adjusted by adding water after weighing the pot.

### 3.4. Measurement of Morphological Traits

After the 25th day of exposure to water stress, the forage plants were harvested, and the plant height, number of tillers, number of leaves, leaf area, root volume, and dry matter of the plant parts were measured. The roots were put in a 1.0 mm mesh sieve and washed under running tap water to remove the adhered soil. The plants were separated into leaves, stems, and roots, oven-dried at 65 °C for three days, and then weighed. The total dry matter was obtained from all seedling parts (leaves, stems, and roots).

The plant height was determined from the soil surface until the insertion of the +1 leaf using a tape measure. The number of tillers or leaves was obtained from their count. Green leaves were detached from plants, and the leaf area was determined using Equation (1), as proposed by Benincasa [36]. Fifteen leaf discs (15.0 cm^2^) detached from the basal, median, and apical leaves constituted the collected sample. Root volume was determined by water displacement method using a 1000 mL graduated cylinder.

LA = [(LAs × LTDM)/DMs],(1)

where LAs is the leaf area of the collected sample, LTDM is the leaf total dry matter, and DMs is the dry matter of the collected sample.

### 3.5. Calculation of Water Stress Tolerance Indices

In this study, 12 water stress tolerance indices proposed by several researchers [37,38,39,40,41,42,43,44] were used to evaluate the forage production response (i.e., shoot biomass production) of the nine forage cultivars grown under a high soil water regime (non-stressful conditions) and under middle and low soil water regimes (i.e., moderate and severe water stress). Forage biomass production data recorded for each forage cultivar in each soil water regime were used to calculate water stress tolerance indices. The 12 water stress tolerance indices used in this study are shown in Table 8.

### 3.6. Statistical Analysis

The data were previously submitted to statistical hypothesis tests to verify the homogeneity of variances (Levene test; *p* > 0.05) and normality of residues (Shapiro–Wilk test; *p* > 0.05). Then, the data were subjected to analysis of variance (ANOVA), and when significant, the means were compared by the Scott–Knott test at the 0.05 confidence level. This analysis was performed using Sisvar^®^ version 5.6 software for Windows (Statistical Analysis Software, UFLA, Lavras, MG, Brazil).

Statistical correlations based on Pearson’s correlation networks (threshold set at 0.60, *p* < 0.05) were performed between the morphological traits of tropical forage grasses. A correlation network was used to graphically illustrate Pearson’s correlation analyses, in which the proximity between the nodes is proportional to the absolute correlation values between the morphological traits. The bands’ relative thickness and color density indicate the strength of Pearson’s correlation coefficients, and the color of each band indicates a positive or negative correlation (red for negative and green for positive).

Canonical correlation analysis (CCA) was used to study the interrelationships between sets (vectors) of independent (forage grass cultivars) and dependent (morphological traits) variables for each soil water regime (high, middle, and low). These analyses were performed using Rbio software version 140 for Windows (Rbio Software, UFV, Viçosa, MG, Brazil).

The identification of water-stress-tolerant forage grass cultivars was performed based on all stress tolerance indices using the ranking method proposed by Farshadfar et al. [21] and improved by Zuffo et al. [45]. In this method, the forage grass cultivars with the highest values for the tolerance indices Y_P_, Y_S_, MP, YSI, DI, STI, GMP, YI, k_1_STI, k_2_STI, SSPI, ATI, and HM received a ranking score of 1. Similarly, the grass cultivars with the lowest values for the TOL tolerance index were assigned a ranking score equal to 1.

The mean ranking score ($\overset{–}{R}$) and the rank standard deviation (RSD) were calculated for all water stress tolerance indices of the nine forage grass cultivars under middle or low soil water regime. The discrimination of forage grass cultivars regarding their tolerance degree to water stress was performed based on the mean ranking score of each grass cultivar, considering the values of the quartiles that divide the nine possible ranking positions (i.e., nine forage grass cultivars) into four equal parts as idealized by Zuffo et al. [45]. A cultivar with a mean rank ($\overset{–}{R}$) lower than the value of the first quartile (<3.0 points) is classified as tolerant (T); a cultivar with an $\overset{–}{R}$ between the first and second quartiles (3.1 to 5.0 points) is classified as moderately tolerant (MT); a cultivar with an $\overset{–}{R}$ between the value of the second and third quartiles (5.1 to 7.0 points) is classified as moderately sensitive (MS); and the group of water-stress-sensitive (S) cultivars is represented by grass cultivar with an $\overset{–}{R}$ higher than the value of the third quartile (≥7.1 points).

## 4. Conclusions

Soil water stress decreased leaf area, plant height, tillering capacity, root volume, and shoot and root dry matter production in most cultivars, with varying degrees of reduction among tropical forage grasses. *Panicum maximum* plants (cv. Mombaça and cv. Tanzânia) grown under controlled greenhouse conditions were identified as tolerant to moderate water stress, whereas the cultivars Aruana and Mombaça of *P. maximum* were identified as tolerant to severe water stress. *Panicum maximum* cv. Mombaça has greater adaptability and stability of shoot biomass production when grown under greenhouse conditions and subjected to middle and low soil water regimes; therefore, this forage grass should be tested under field conditions to confirm its forage production potential for cultivation in tropical regions with the occurrence of water stress. The MP, DI, STI, GMP, YI, k_2_STI, and HM tolerance indices were the most suitable for identifying forage grass cultivars with greater water stress tolerance and a high potential for shoot biomass production under low soil water regime.

## Figures and Tables

**Table 1:** Summary of analysis of variance for morphological traits of tropical forage grass cultivars under the effect of soil water regimes.

Causes of Variation	Probability > F							
	PH	NT	NL	LA	SDM	RDM	TDM	RV
Forage cultivar (C)	<0.01	<0.01	<0.01	<0.01	<0.01	<0.01	<0.01	<0.01
Soil water regime (W)	<0.01	<0.01	<0.01	<0.01	<0.01	<0.01	<0.01	<0.01
C × W	<0.01	0.914	<0.01	0.045	0.518	0.020	0.029	<0.01
CV (%)	13.65	16.41	18.61	17.82	15.99	20.66	15.70	21.92

PH: plant height; NT: number of tillers; NL: number of leaves; LA: leaf area; SDM: shoot dry matter; RDM: root dry matter; TDM: total dry matter; RV: root volume. CV: coefficient of variation.

**Table 2:** Plant height, number of green leaves, and leaf area of nine tropical forage grass cultivars grown under different soil water regimes.

Forage Grass Cultivar	Soil Water Regime		
	High	Middle	Low
	Plant Height (cm)		
*U. brizantha* cv. BRS Piatã	74.0 ± 5.0 bA	63.7 ± 8.1 bB	53.3 ± 4.7 bC
*U. brizantha* cv. Marandu	62.7 ± 10.1 cA	51.3 ± 4.8 cA	50.0 ± 2.5 bA
*U. brizantha* cv. Xaraés	62.0 ± 9.9 cA	56.0 ± 2.0 cA	50.7 ± 2.4 bA
*U. ruziziensis* cv. Comum	47.7 ± 6.4 cA	47.3 ± 2.4 cA	43.3 ± 2.9 bA
*P. glaucum* cv. ADR 300	154.1 ± 5.3 aA	124.6 ± 5.1 aB	86.2 ± 3.3 aC
*P. maximum* cv. Aruana	72.7 ± 3.7 bA	71.0 ± 7.5 bA	71.3 ± 1.8 aA
*P. maximum* cv. Mombaça	79.7 ± 6.4 bA	62.7 ± 4.1 bB	62.0 ± 2.0 bB
*P. maximum* cv. Tanzânia	68.7 ± 5.8 bA	63.7 ± 5.3 bA	63.7 ± 1.3 bA
*P. atratum* cv. Pojuca	62.0 ± 9.9 cA	52.3 ± 1.4 cA	52.7 ± 3.7 bA
	**Number of leaves per plant**		
*U. brizantha* cv. BRS Piatã	40 ± 1 dA	33 ± 3 bA	20 ± 1 bA
*U. brizantha* cv. Marandu	41 ± 4 dA	32 ± 3 bA	24 ± 1 bA
*U. brizantha* cv. Xaraés	36 ± 2 dA	26 ± 1 bA	18 ± 1 bA
*U. ruziziensis* cv. Comum	119 ± 6 bA	88 ± 2 aB	51 ± 3 aC
*P. glaucum* cv. ADR 300	26 ± 2 dA	21 ± 1 bA	15 ± 2 bA
*P. maximum* cv. Aruana	75 ± 5 cA	47 ± 5 bB	38 ± 3 bB
*P. maximum* cv. Mombaça	52 ± 6 dA	30 ± 2 bB	23 ± 3 bC
*P. maximum* cv. Tanzânia	53 ± 2 dA	32 ± 1 bA	32 ± 5 bA
*P. atratum* cv. Pojuca	195 ± 20 aA	94 ± 19 aB	70 ± 10 aC
	**Leaf area (dm^2^/plant)**		
*U. brizantha* cv. BRS Piatã	16.9 ± 1.2 bA	10.2 ± 0.3 bB	7.6 ± 0.5 aB
*U. brizantha* cv. Marandu	20.8 ± 2.2 bA	17.5 ± 5.1 aA	9.9 ± 0.9 aB
*U. brizantha* cv. Xaraés	21.0 ± 1.2 bA	14.8 ± 1.5 aB	8.8 ± 1.5 aC
*U. ruziziensis* cv. Comum	31.6 ± 6.8 aA	18.9 ± 2.9 aB	11.9 ± 1.6 aC
*P. glaucum* cv. ADR 300	1.6 ± 0.2 dA	1.4 ± 0.1 bA	1.3 ± 0.2 bA
*P. maximum* cv. Aruana	28.9 ± 1.6 aA	17.0 ± 2.0 aB	11.9 ± 1.8 aB
*P. maximum* cv. Mombaça	28.6 ± 2.7 aA	18.8 ± 3.7 aB	12.0 ± 1.3 aC
*P. maximum* cv. Tanzânia	23.3 ± 2.3 bA	21.1 ± 2.3 aA	12.9 ± 0.8 aB
*P. atratum* cv. Pojuca	10.8 ± 1.6 cA	5.5 ± 0.8 bA	4.4 ± 1.3 bA

Means followed by distinct lowercase letters for the forage grass cultivars (in the column) or distinct uppercase letters for the soil water regimes (in the line) show significant differences (Scott–Knott test, *p* ≤ 0.05). Values represent the mean ± mean standard error.

**Table 3:** Root dry matter, total dry matter, and root volume of nine tropical forage grass cultivars grown under different soil water regimes.

Forage Grass Cultivar	Soil Water Regimes		
	High	Middle	Low
	**Root dry matter (g plant^–1^)**		
*U. brizantha* cv. BRS Piatã	23.3 ± 3.4 aA	15.0 ± 5.5 aB	5.5 ± 1.0 aC
*U. brizantha* cv. Marandu	19.5 ± 1.3 aA	12.5 ± 1.2 aB	7.5 ± 1.0 aB
*U. brizantha* cv. Xaraés	14.0 ± 1.4 bA	12.2 ± 1.1 aA	8.8 ± 1.1 aA
*U. ruziziensis* cv. Comum	22.8 ± 1.0 aA	14.4 ± 0.3 aB	7.1 ± 0.6 aC
*P. glaucum* cv. ADR 300	5.4 ± 0.6 cA	5.2 ± 0.4 bA	3.6 ± 0.2 aA
*P. maximum* cv. Aruana	17.5 ± 3.9 aA	8.3 ± 1.1 bB	1.2 ± 0.1 aC
*P. maximum* cv. Mombaça	28.4 ± 6.7 aA	13.1 ± 0.6 aB	8.3 ± 0.9 aB
*P. maximum* cv. Tanzânia	21.0 ± 3.6 aA	15.0 ± 3.4 aB	12.3 ± 1.2 aB
*P. atratum* cv. Pojuca	7.0 ± 1.5 cA	3.6 ± 0.7 bA	2.9 ± 0.3 aA
	**Total dry matter (g plant^–1^)**		
*U. brizantha* cv. BRS Piatã	55.6 ± 4.3 bA	36.0 ± 1.3 bB	23.0 ± 5.9 bC
*U. brizantha* cv. Marandu	50.7 ± 1.6 bA	35.3 ± 2.0 bB	23.2 ± 1.7 bC
*U. brizantha* cv. Xaraés	42.4 ± 3.9 cA	32.5 ± 1.8 bB	22.6 ± 0.7 bC
*U. ruziziensis* cv. Comum	56.8 ± 1.0 bA	45.9 ± 3.5 aB	26.1 ± 1.3 aC
*P. glaucum* cv. ADR 300	33.3 ± 0.3 cA	25.3 ± 1.1 cB	17.7 ± 0.2 bB
*P. maximum* cv. Aruana	58.1 ± 4.3 bA	34.0 ± 7.4 bB	26.8 ± 0.2 aB
*P. maximum* cv. Mombaça	69.3 ± 7.1 aA	42.1 ± 1.2 aB	29.9 ± 0.7 aC
*P. maximum* cv. Tanzânia	56.3 ± 4.8 bA	43.2 ± 4.3 aB	33.7 ± 1.4 aB
*P. atratum* cv. Pojuca	34.4 ± 1.8 cA	18.0 ± 4.2 cB	17.3 ± 1.2 bB
	**Root volume (cm^3^ plant^–1^)**		
*U. brizantha* cv. BRS Piatã	88.9 ± 19.8 dA	64.4 ± 6.8 aB	38.9 ± 5.6 aC
*U. brizantha* cv. Marandu	106.7 ± 3.3 cA	61.8 ± 6.7 aB	37.8 ± 4.0 aB
*U. brizantha* cv. Xaraés	94.0 ± 6.8 cA	58.9 ± 9.5 aB	32.9 ± 0.4 aC
*U. ruziziensis* cv. Comum	154.4 ± 14.6 aA	85.0 ± 8.7 aB	33.2 ± 1.9 aC
*P. glaucum* cv. ADR 300	53.3 ± 5.7 dA	31.7 ± 1.0 bB	23.3 ± 1.9 aB
*P. maximum* cv. Aruana	113.0 ± 3.5 cA	85.0 ± 16.0 aB	38.9 ± 2.2 aC
*P. maximum* cv. Mombaça	162.8 ± 11.4 aA	71.1 ± 8.7 aB	52.2 ± 1.1 aB
*P. maximum* cv. Tanzânia	133.3 ± 19.0 bA	83.3 ± 3.8 aB	58.9 ± 6.8 aC
*P. atratum* cv. Pojuca	64.4 ± 2.2 dA	22.1 ± 9.0 bB	21.1 ± 4.0 aB

Means followed by distinct lowercase letters for the forage grass cultivars (in the column) or distinct uppercase letters for the soil water regimes (in the line) show significant differences (Scott Knott test, *p* ≤ 0.05). Values represent the mean ± mean standard error.

**Table 4:** Average value of tiller number and shoot dry matter of tropical forage grasses affected by cultivars and soil water regimes.

Causes of Variation	Number of Tillers (Units)	Shoot Dry Matter (g plant^–1^)
**Forage grass cultivars**		
*U. brizantha* cv. BRS Piatã	10.8 ± 0.8 d	20.8 ± 2.6 b
*U. brizantha* cv. Marandu	12.3 ± 0.9 d	23.2 ± 2.3 b
*U. brizantha* cv. Xaraés	11.1 ± 0.7 d	20.8 ± 2.2 b
*U. ruziziensis* cv. Comum	26.1 ± 3.0 a	28.2 ± 2.6 a
*P. glaucum* cv. ADR 300	4.2 ± 0.3 e	20.8 ± 2.0 b
*P. maximum* cv. Aruana	18.4 ± 1.2 b	30.6 ± 3.4 a
*P. maximum* cv. Mombaça	15.8 ± 1.0 c	30.5 ± 3.1 a
*P. maximum* cv. Tanzânia	15.6 ± 1.0 c	28.3 ± 2.2 a
*P. atratum* cv. Pojuca	19.8 ± 1.4 b	18.2 ± 2.6 b
**Soil water regimes**		
High	16.4 ± 1.2 a	33.1 ± 1.1 a
Middle	15.6 ± 1.6 a	23.6 ± 1.4 b
Low	12.6 ± 1.1 b	17.8 ± 0.8 c

Means followed by distinct lowercase letters for the forage grass cultivar and soil water regime (in the column) show significant differences (Scott Knott test, p).

**Table 5:** Shoot biomass production and stress tolerance indices of the nine forage grass cultivars under middle or low soil water regimes.

Forage Grass Cultivar	Y_P_^†^	Y_S_^††^	Water Stress Tolerance Indices											
			TOL	MP	YSI	DI	STI	GMP	YI	k_1_STI	k_2_STI	SSPI	ATI	HM
	**Middle soil water regime (MSW)**													
*U. brizantha* cv. BRS Piatã	32.3 b	21.0 a	11.3 a	26.6 b	0.65 a	0.58 a	0.62 b	26.0 b	0.89 a	0.95 b	0.89 a	23.9 a	210.4 a	25.4 b
*U. brizantha* cv. Marandu	31.3 b	22.8 a	8.5 a	27.0 b	0.73 a	0.71 a	0.65 b	26.7 b	0.96 a	0.89 b	0.96 a	17.9 a	161.1 a	26.3 b
*U. brizantha* cv. Xaraés	28.4 b	20.2 a	8.2 a	24.3 b	0.72 a	0.61 a	0.53 b	240 b	0.86 a	0.75 b	0.86 a	17.3 a	143.5 a	23.6 b
*U. ruziziensis* cv. Comum	34.0 b	28.1 a	5.9 a	31.1 a	0.83 a	0.99 a	0.88 a	30.9 a	1.19 a	1.06 b	1.19 a	12.4 a	131.5 a	30.8 a
*P. glaucum* cv. ADR 300	28.0 b	20.1 a	7.8 a	24.0 b	0.72 a	0.61 a	0.51 b	23.7 b	0.85 a	0.71 b	0.85 a	16.5 a	132.6 a	23.4 b
*P. maximum* cv. Aruana	40.5 a	25.7 a	14.8 a	33.1 a	0.63 a	0.82 a	0.95 a	31.3 a	1.09 a	1.50 a	1.09 a	31.4 a	270.3 a	29.8 a
*P. maximum* cv. Mombaça	41.0 a	29.0 a	12.0 a	35.0 a	0.72 a	0.89 a	1.08 a	34.3 a	1.23 a	1.57 a	1.23 a	25.4 a	305.4 a	33.7 a
*P. maximum* cv. Tanzânia	35.3 a	28.2 a	7.1 a	31.8 a	0.80 a	0.96 a	0.91 a	31.5 a	1.19 a	1.14 b	1.19 a	15.1 a	157.2 a	31.2 a
*P. atratum* cv. Pojuca	27.4 b	14.3 a	13.0 a	20.8 b	0.53 a	0.36 a	0.36 b	19.5 b	0.61 a	0.68 b	0.61 a	27.6 a	169.6 a	18.3 b
Mean	5.11	4.41	1.83	4.68	0.06	0.19	0.22	4.80	0.25	0.33	0.25	5.19	55.79	4.91
CV (%)	15.41	24.93	11.88	18.43	11.02	35.71	40.32	19.88	24.93	31.60	24.93	11.88	27.64	21.38
	**Low soil water regime (LSW)**													
*U. brizantha* cv. BRS Piatã	32.3 b	14.8 d	17.5 a	23.6 c	0.46 a	0.39 d	0.44 d	21.9 c	0.84 d	0.95 b	0.84 c	49.4 a	203.9 b	20.3 d
*U. brizantha* cv. Marandu	31.3 b	15.6 d	15.6 a	23.4 c	0.50 a	0.44 d	0.45 d	22.1 c	0.88 d	0.89 b	0.88 c	44.2 a	184.6 b	20.8 d
*U. brizantha* cv. Xaraés	28.4 b	13.8 d	14.6 a	21.1 c	0.49 a	0.39 d	0.36 d	19.8 c	0.78 d	0.75 b	0.78 c	41.2 a	156.4 b	18.5 d
*U. ruziziensis* cv. Comum	34.0 b	19.0 c	15.0 a	26.5 b	0.56 a	0.61 c	0.59 c	25.4 b	1.08 c	1.06 b	1.08 c	42.3 a	204.6 b	24.4 c
*P. glaucum* cv. ADR 300	28.0 b	14.2 d	13.8 a	21.1 c	0.51 a	0.41 d	0.36 d	19.9 c	0.80 d	0.71 b	0.80 c	39.1 a	146.9 b	18.8 d
*P. maximum* cv. Aruana	40.5 a	25.6 a	14.9 a	33.1 a	0.63 a	0.91 a	0.95 a	32.2 a	1.45 a	1.50 a	1.45 a	42.3 a	257.0 a	31.4 a
*P. maximum* cv. Mombaça	41.0 a	21.6 b	19.4 a	31.3 a	0.53 a	0.65 c	0.82 b	29.7 a	1.22 b	1.57 a	1.22 b	54.7 a	315.7 a	28.3 b
*P. maximum* cv. Tanzânia	35.3 a	21.4 b	13.9 a	28.4 b	0.61 a	0.74 b	0.69 b	27.5 b	1.21 b	1.14 b	1.21 b	39.3 a	204.7 b	26.7 c
*P. atratum* cv. Pojuca	27.4 b	13.0 d	14.3 a	20.2 c	0.48 a	0.36 d	0.32 d	18.8 c	0.74 d	0.68 b	0.74 c	40.6 a	142.8 b	17.5 d
Mean	5.11	4.88	3.04	4.76	0.09	0.21	0.24	4.78	0.21	0.33	0.21	6.43	62.54	4.86
CV (%)	15.41	20.98	30.89	16.88	12.90	28.55	33.69	17.35	20.98	31.60	20.98	30.89	33.47	18.03

^†^ Y_P_ represents the shoot biomass production (in grams per plant) of forage grasses grown under high soil water regime (non-stressful condition). ^††^ Y_S_ represents the shoot biomass production (in grams per plant) of forage grasses exposed to water stress conditions (middle or low soil water regime). Means followed by distinct lower case letters for the forage grass cultivar and stress tolerance indices (in the column) show significant differences (Scott Knott test, p). CV: Coefficient of variation. TOL: Tolerance, MP: Mean productivity, YSI: Yield stability index, DI: Drought resistance index, STI: GMP: Stress tolerance index, GMP: Geometric mean productivity, YI: Yield index, k_1_STI: Modified stress tolerance (k1), k_2_STI: Modified stress tolerance (k2), SSPI: Stress susceptibility percentage index, ATI: Abiotic tolerance index, HM: Harmonic mean.

**Table 6:** Rank, mean rank ($\overset{–}{R}$), and standard deviation of ranks (SD) of water stress tolerance indices of the nine forage grass cultivars under middle or low soil water regimes.

Forage Grass Cultivar	Y_P_	Y_S_	Stress Tolerance Indices												$\overset{–}{R}$ (±SD)	Tolerance Level ^†^
			TOL	MP	YSI	DI	STI	GMP	YI	k_1_STI	k_2_STI	SSPI	ATI	HM		
	Middle Soil Water Regime															
*U. brizantha* cv. BRS Piatã	5	6	6	6	7	8	6	6	6	5	6	4	3	6	5.7 (±0.8)	MS
*U. brizantha* cv. Marandu	6	5	5	5	3	5	5	5	5	6	5	5	5	5	5.0 (±0.3)	MT
*U. brizantha* cv. Xaraés	7	7	4	7	6	7	7	7	7	7	7	6	7	7	6.6 (±0.6)	MS
*U. ruziziensis* cv. Comum	4	3	1	4	1	1	4	4	3	4	3	9	9	3	3.8 (±1.6)	MT
*P. glaucum* cv. ADR 300	8	8	3	8	5	6	8	8	8	8	8	7	8	8	7.2 (±1.1)	S
*P. maximum* cv. Aruana	2	4	9	2	8	4	2	3	4	2	4	1	2	4	3.6 (±1.6)	MT
*P. maximum* cv. Mombaça	1	1	7	1	4	3	1	1	1	1	1	3	1	1	1.9 (±1.3)	T
*P. maximum* cv. Tanzânia	3	2	2	3	2	2	3	2	2	3	2	8	6	2	3.0 (±1.1)	T
*P. atratum* cv. Pojuca	9	9	8	9	9	9	9	9	9	9	9	2	4	9	8.1 (±1.5)	S
	**Low Soil Water Regime**															
*U. brizantha* cv. BRS Piatã	5	6	8	5	9	7	6	6	6	5	6	2	5	4	5.7 (±1.2)	MS
*U. brizantha* cv. Marandu	6	5	7	6	6	5	5	5	5	6	5	3	6	5	5.4 (±0.7)	MS
*U. brizantha* cv. Xaraés	7	8	4	7	7	8	8	8	8	7	8	6	7	2	6.8 (±1.2)	MS
*U. ruziziensis* cv. Comum	4	4	6	4	3	4	4	4	4	4	4	4	4	6	4.2 (±0.5)	MT
*P. glaucum* cv. ADR 300	8	7	1	8	5	6	7	7	7	8	7	9	8	3	6.5 (±1.6)	MS
*P. maximum* cv. Aruana	2	1	5	1	1	1	1	1	1	2	1	5	2	9	2.4 (±1.7)	T
*P. maximum* cv. Mombaça	1	2	9	2	4	3	2	2	2	1	2	1	1	8	2.9 (±1.8)	T
*P. maximum* cv. Tanzânia	3	3	2	3	2	2	3	3	3	3	3	8	3	7	3.4 (±1.2)	MT
*P. atratum* cv. Pojuca	9	9	3	9	8	9	9	9	9	9	9	7	9	1	7.8 (±1.8)	S

^†^ T refers to a water-stress-tolerant cultivar with a mean rank ($\overset{–}{R}$) score of 1 to 3.0; MT, moderately tolerant cultivar with an $\overset{–}{R}$ score of 3.1 to 5.0; MS, moderately sensitive cultivar with an $\overset{–}{R}$ score of 5.1 to 7.0; and S, water-stress-sensitive cultivar with an $\overset{–}{R}$ score of 7.1 to 9. TOL: Tolerance, MP: Mean productivity, YSI: Yield stability index, DI: Drought resistance index, STI: GMP: Stress tolerance index, GMP: Geometric mean productivity, YI: Yield index, k_1_STI: Modified stress tolerance (k1), k_2_STI: Modified stress tolerance (k2), SSPI: Stress susceptibility percentage index, ATI: Abiotic tolerance index, HM: Harmonic mean.

**Table 7:** Some characteristics of the nine tropical forage grass cultivars used in this study.

Forage Grass Cultivar	Common Name	Cultivar	Growth Habit	Soil Fertility Requirement	Forage Yield	Drought Tolerance
*Urochloa brizantha*	Palisade grass	BRS Piatã	Semierect	Medium	High	High
*Urochloa brizantha*	Palisade grass	Marandu	Erect	Medium	Medium	Medium
*Urochloa brizantha*	Palisade grass	Xaraés	Semierect	Medium	High	Medium
*Panicum maximum*	Guinea grass	Aruana	Erect	Medium/High	High	Medium/Low
*Panicum maximum*	Guinea grass	Mombaça	Erect	Medium/High	High	Medium/Low
*Panicum maximum*	Guinea grass	Tanzânia	Erect	Medium/High	High	Medium/Low
*Pennisetum glaucum*	Pearl millet	ADR 300	Erect	Medium	High	Medium/High
*Urochloa ruziziensis*	Ruzigrass	Comum	Semierect	Medium/High	High	Low
*Paspalum atratum*	Atratum	Pojuca	Erect	Low	High	Low

Source: Fonseca and Martusello [5].

**Table 8:** Stress tolerance indices used to assess water stress tolerance of nine tropical forage grass cultivars grown under different soil water regimes.

Water Stress Tolerance Index	Equation ^†^	Reference
1. Tolerance	TOL = Y_P_ − Y_S_	[37]
2. Mean productivity	MP = (Y_S_ + Y_P_)/2	[37]
3. Yield stability index	YSI = Y_S_/Y_P_	[38]
4. Drought resistance index	DI = [Y_S_ × (Y_S_/Y_P_)]/Ȳ_S_	[39]
5. Stress tolerance index	STI = (Y_S_ × Y_P_)/(Ȳ_P_)2	[40]
6. Geometric mean productivity	GMP = √(Y_S_ × Y_P_)	[40]
7. Yield index	YI = Y_S_/Ȳ_S_	[41]
8. Modified stress tolerance (k_1_)	k_1_STI = Y_P_^2^/Ȳ_P_^2^	[42]
9. Modified stress tolerance (k_2_)	k_2_STI = Y_S_^2^/Ȳ_S_^2^	[42]
10. Stress susceptibility percentage index	SSPI = [(Y_P_ − Y_S_)/2 × Ȳ_P_] × 100	[43]
11. Abiotic tolerance index	ATI = [(Y_P_–Y_S_)/(Ȳ_P_/Ȳ_S_)] × √(Y_P_ × YS)	[43]
12. Harmonic mean	HM = [2 × (Y_S_ × Y_P_)]/(Y_S_ + Y_P_)	[44]

^†^ In the equations above, Y_P_ and Y_S_ represent the forage production of grasses grown under high soil water regime (non-stressful condition) and under middle or low soil water regimes (moderate or severe water stress) for each forage grass cultivar, respectively, whereas Ȳ_P_ and Ȳ_S_ represent the average forage production of all grass cultivars under high soil water regime and under middle or low soil water regimes, respectively.

## Data Availability

All available data can be obtained by contacting the corresponding author.

## Abbreviations

FC: field capacity; HSW: high soil water; ICLS: integrated crop–livestock system; LA: leaf area; LSW: low soil water; MSW: middle soil water; NT: number of tillers; NL: number of leaves; PH: plant height; RDM: root dry matter; RV: root volume; SDM: shoot dry matter; TDM: total dry matter.
